# Effect of Elastic Abdominal Binder on Pain and Functional Recovery Following Gynecologic Cancer Surgery: A Randomized Controlled Trial

**DOI:** 10.3390/medicina57050481

**Published:** 2021-05-12

**Authors:** Nopwaree Chantawong, Kittipat Charoenkwan

**Affiliations:** Division of Gynecologic Oncology, Department of Obstetrics and Gynecology, Faculty of Medicine, Chiang Mai University, Chiang Mai 50200, Thailand; meowwcool@gmail.com

**Keywords:** elastic abdominal binder, functional recovery, gynecologic neoplasm, postoperative pain, six-minute walk test

## Abstract

*Background and Objectives*: Clinicians have been using elastic abdominal binder for stabilizing incision site after major abdominal surgery. However, the benefits of that practice have never been formally assessed. The aim of this study was to examine the effects of the use of elastic abdominal binder on postoperative pain and recovery of gynecologic cancer patients. *Materials and Methods*: One-hundred and nine women diagnosed with cervical, endometrial, or ovarian cancer, who underwent open abdominal surgery were assigned randomly into two groups: intervention (56 patients) and control (53 patients). The women in the intervention group applied abdominal binder from postoperative day 1. For the control group, the women did not wear the binder or similar devices. The primary outcomes were pain and functional recovery. Subgroup analysis on participants age ≥ 50 was also performed. *Results*: For the entire study cohort, the baseline, postoperative day 1, and postoperative day 2 pain scores in the intervention group were significantly lower than the control group. However, there was no significant difference between the groups for postoperative day 3 pain score and for the change in pain scores from the baseline value. Of note, the age ≥ 50 subgroup represented a more balanced cohort with comparable baseline pain scores between the study groups. For this population, the pain scores for postoperative day 1–3 were significantly lower in the intervention group. The intervention group had a longer six-minute walking distance on postoperative day 3 with a trend toward a smaller difference in the day 3 distance from the baseline. *Conclusions*: The potential benefits of abdominal binder use in reducing postoperative pain and improving functional recovery after open gynecologic cancer surgery could be demonstrated only in those age ≥ 50.

## 1. Introduction

Surgery is a major component of multidisciplinary care for gynecologic cancers [1]. The primary surgical approach provides an opportunity for the therapeutic removal of the organs affected by the cancer and for the complete assessment of the extent of the cancer spread [2,3]. However, the surgical management is often invasive, and frequently associated with significant perioperative morbidity and delayed postoperative functional recovery. This is particularly true when an open laparotomy approach is needed for the adequate resection of a large primary tumor or extensive debulking of an intraperitoneal cancer.

Abdominal binder is a broad elastic band that is applied around the patient’s torso at the level of the lower abdomen to provide support to the surgical incision after surgery. Clinicians have been using the binder for stabilizing the incision site, relieving incisional pain, improving mobility, and preventing wound complications such as seroma and hematoma [4]. At our department, surgeons have offered elastic abdominal binder to gynecologic cancer patients following surgery for several years. However, the benefit of that practice has not been formally assessed.

High-quality data on the effectiveness of abdominal binder use in this particular group of patients is lacking [4,5,6]. Earlier randomized controlled studies on abdominal binder use for promoting postoperative recovery examined patients who had gastrointestinal surgery. Benefits on pain relief, physical function improvement, and psychological distress reduction were evidenced in the Cheifetz et al. [5] and Arici et al. [7] studies. However, none of these advantages were demonstrated in a similar study by Olsen et al. [8]. Likewise, data from randomized controlled trials on benefits of the binder use following cesarean delivery were conflicting [9,10,11,12]. In the only study that assessed the role of abdominal binder use after gynecologic surgery [13], the authors observed increased number of ambulatory events in the first 24 h associated with the binder use but only in those who had vertical incision and age ≥ 50. No other benefits were found. Of note, patients with gynecologic cancers made up merely 37% of the participants of that study. Therefore, the Enhanced Recovery After Surgery (ERAS) initiative for gynecologic cancer surgery, which is increasingly adopted worldwide, has not included the use of this device in its perioperative protocol [14]. 

The aim of this study was to examine the effects of elastic abdominal binder use on the postoperative recovery of gynecologic cancer patients focusing on postoperative pain, functional recovery, postoperative complications, and quality of life.

## 2. Materials and Methods

### 2.1. Study Population

We invited women aged 18–65 years, diagnosed with cervical, endometrial, or ovarian cancer, who underwent open major abdominal surgery at our institution from April 2018 to May 2019 to participate in this parallel group randomized controlled trial. For participants with cervical cancer, the operative procedures performed included radical (type B or C) hysterectomy and pelvic lymphadenectomy with/without para-aortic lymphadenectomy. For those with endometrial or ovarian cancers, total (type A) hysterectomy, bilateral salpingo-oophorectomy, pelvic and para-aortic lymphadenectomy, omentectomy, intraperitoneal biopsies, and tumor debulking (for advanced disease) were performed. General anesthesia induced by nitrous oxide, intravenous midazolam and fentanyl was employed in all patients. The anesthesia was maintained by inhaled anesthetics (desflurane/sevoflurane) and/or additional fentanyl. Combined general anesthesia with epidural anesthesia was used at the discretion of the attending anesthesiologist. The exclusion criteria were intraoperative accidental injury to urinary or gastrointestinal organs, the need for ostomy, postoperative admission to intensive care unit, postoperative intraperitoneal drain placement, unable to understand and follow oral/written instructions, and severe neuromuscular or circulatory disorders. The protocol of this study was registered with ClinicalTrials.gov (ID NCT03818633).

### 2.2. Study Intervention 

At the time of procedure completion, the study coordinator randomly assigned the participants into two study groups, abdominal binder (intervention) and no binder (control). The computer-generated stratified randomization sequence by primary site of cancer was employed. The allocation assignments were placed in sequentially numbered sealed opaque envelopes to ensure allocation concealment.

The day of operation was considered postoperative day 0. For the intervention group, each participant was fitted with an elastic abdominal binder starting on postoperative day 1 at 7 a.m. The participants were instructed and assisted to wear the binder snugly around their torso, on top of their hospital robe, with the incision positioned at the center of the binder band. We encouraged them to keep the binder on at all times during their hospital stay. Nevertheless, occasional breaks from binder wearing were allowed at the participants’ convenience. For the control group, the participants did not wear abdominal binder or similar devices. Standard postoperative care protocol and medication were provided uniformly to both study groups. All participants were encouraged to ambulate within 6 h after surgery and offered sips of water when fully awake. On postoperative day 1, liquid diet was started in the morning and, if tolerated, progressed to soft diet for the next meal and regular diet thereafter if desired. Regarding postoperative pain medication, the patients received either intravenous parecoxib every 12 h with intravenous morphine for breakthrough pain or patient-controlled analgesia (PCA) at the discretion of the attending anesthesiologist on the day of surgery and postoperative day 1 and 2. Oral diclofenac or tramadol in combination with acetaminophen was used thereafter as needed. Foley catheter was usually removed on postoperative day 1 given reasonable level of self-care and adequate urine output.

Hospital discharge criteria included stable vital signs, no active ongoing postoperative complications, pain control with oral analgesia, good tolerance of oral soft diet, and return of the ability to carry on daily self-care.

### 2.3. Study Outcomes

Primary outcomes for this study were postoperative pain and functional recovery. Secondary outcomes were quality of life and postoperative complications.

#### 2.3.1. Primary Outcomes

##### Pain

We asked each participant to rate her visual analogue scale pain score at 6:00 a.m. on postoperative day 1. This was used as baseline postoperative pain score. Thereafter, we assessed pain scores twice daily at 10:00 a.m. and 6:00 p.m. The average of these two pain scores represented pain score for that day. The number and dosage of postoperative analgesics needed were also recorded. The difference in the pain score from baseline was calculated each day.

##### Functional Recovery

Functional recovery was assessed by using the mobility aspect of the six-minute walk test. This is one of the best established, simplest, best tolerated, and most reflective technique of measuring daily activity among the methods for the evaluation of functional capacity [15]. We performed the six-minute walk tests at baseline one day before surgery and in the morning of postoperative day 2 and 3 on a long, straight corridor with a hard surface as per the American Thoracic Society consensus conference statement guidelines [16]. The difference in postoperative day 2 and 3 walking distance from the baseline was calculated.

#### 2.3.2. Secondary Outcomes

##### Quality of Life

For the quality-of-life assessment, we administered the EuroQol Group’s EQ-5D-5L questionnaire on postoperative day 3 [17]. The quality-of-life aspects on mobility, self-care, usual activities, pain/discomfort, and anxiety were evaluated. For each aspect, we asked the participants to rate their experience as no problems (score 1), mild problems (score 2), moderate problems (score 3), severe problems (score 4), and most severe problems (score 5). Furthermore, we classified the scores of 1 and 2 in any aspects as normal and the scores of 3 to 5 as problem. Also, we requested the participants to rate their overall health status according to a visual analogue scale with 0 representing the worst health imaginable and 100 indicating the best health imaginable.

##### Postoperative Complications

We monitored postoperative complications, which included febrile morbidity, wound complications, and bowel ileus daily. We defined febrile morbidity as having a temperature of 38 degree Celsius or greater on two successive episodes, measured six hours apart using a standard technique, excluding the first 24 h following surgery. Wound complications included hematoma, seroma, and dehiscence. Clinically significant bowel ileus was diagnosed if participants had combined symptoms of severe nausea/vomiting, abdominal distention, and hypoactive bowel sound.

A specially trained registered nurse recorded pain scores, performed the six-minute walk test, and acquired quality of life information through an interview session. The team of attending physicians responsible for gynecologic oncology postoperative recovery ward evaluated postoperative complications.

### 2.4. Sample Size Calculation

We based sample size calculation on the primary outcome of the visual analogue scale pain scores. We chose a 2 cm difference between the study groups in visual analogue scale pain scores as the minimal effect that would be clinically meaningful [18]. We set the alpha value at 0.05 and the power at 90% with standard deviations in visual analogue scale pain scores of each study group derived from a similar study employing abdominal binder after cesarean delivery [11], the sample size was determined to be at least 50 for each group.

### 2.5. Statistical Analysis

Statistical analysis was performed with Stata^®^ program version 15 (StataCorp LP, College Station, TX, USA). Continuous outcome variables (pain scores, 6-min walking distance, and quality of life scores) were expressed as median if the data distribution were skewed and mean if the data distribution were normal. We applied the Mann-Whitney U test or Student’s *t*-test, as appropriate, for hypothesis testing for difference in continuous outcomes between the study groups. Hypothesis testing for categorical outcome variables was performed by using chi square or Fisher’s exact test, as deemed appropriate. A *p*-value of < 0.05 was considered statistically significant.

In addition, subgroup analysis on participants age ≥ 50 was performed given that this is the population that may benefit from earlier mobilization promoted by the binder use. Moreover, the benefit of increased number of ambulatory events in the first 24 h with the binder use was observed only in this particular group of participants in the previous study examining gynecologic patients [13].

## 3. Results

**Figure 1:**
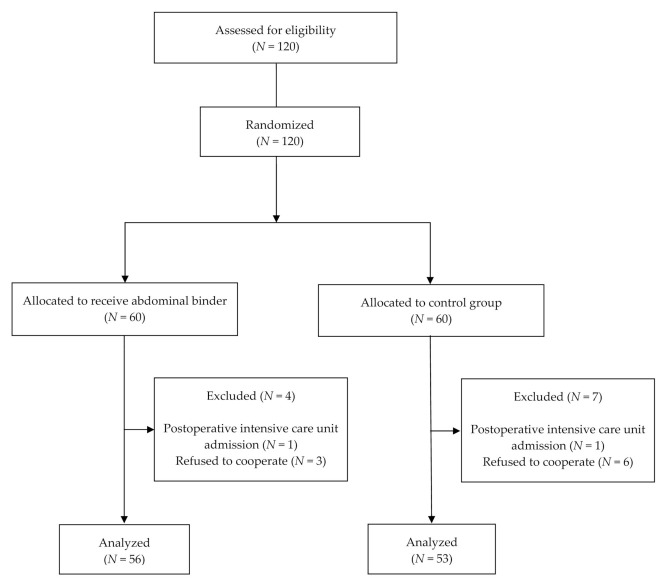
CONSORT flow diagram.

From April 2018 to May 2019, 120 eligible women agreed to participate and were randomly allocated into two groups, intervention (60 women) and control (60 women). Two patients, one from each study group, were excluded due to the need for postoperative intensive care unit admission because of perioperative medical issues (myocardial infarction and congestive heart failure). In addition, nine patients (three in the intervention group and six in the control group) refused to cooperate and requested to exit the study on postoperative day 1 without receiving the assigned intervention. The remaining 109 patients, 56 patients in the intervention group and 53 patients in the control group, participated in the allocated study groups through to the completion of the study (Figure 1).

Table 1 demonstrates clinical and surgical characteristics of the participants. All characteristics were comparable between the study groups. Likewise, for the 67 participants in the age ≥ 50 subgroup (37 in the intervention arm and 30 in the control arm), these baseline characteristics were similar between the two arms. Median age were 58.0 years for the intervention group and 61.5 years for the control group (*p* = 0.07). Mean body mass indices were comparable, 24.7 kg/m^2^ in the intervention group and 25.8 kg/m^2^ in the control group (*p* = 0.26). Median incision length was 15.0 cm for both study groups. Median operative blood loss was 250 mL for the intervention group and 325 mL for the control group (*p* = 0.35). Median operative time was comparable, 177.0 min in the intervention group and 176.5 min in the control group (*p* = 0.69).

Table 2 compares postoperative pain scores between the two study groups. In general, the pain increased from baseline and peaked in postoperative day 1 before gradually decreased in postoperative day 2 and 3. The baseline, postoperative day 1, and postoperative day 2 pain scores for the intervention group were significantly lower than the control group. However, there was no significant difference between the groups in postoperative day 3 pain score and in the change in postoperative day 1–3 pain scores from the baseline. Of note, the age ≥ 50 subgroup represented a more balanced cohort with comparable baseline pain scores between the study groups. For this population, the pain scores on postoperative day 1–3 were significantly lower in the intervention group. Furthermore, the pain scores of those in the intervention group had started to get lower than the baseline since postoperative day 2. This trend became clearer on postoperative day 3.

Table 3 demonstrates postoperative consumption of pain medication. While the morphine consumption was comparable, the parecoxib and acetaminophen were needed significantly less in the intervention group. There was no difference in the rate of patient-controlled analgesia and epidural analgesia use between the groups.

Table 4 summarizes the six-minute walking distance at the baseline level and on postoperative day 2 and 3. The walking distances between groups were not significantly different at the baseline level. Overall, the walking distance dropped significantly from the baseline level, but gradually improved over time. Those assigned to the intervention group had significantly longer six-minute walking distances on postoperative day 3, with a trend toward less difference in the day 3 distance from baseline. The six-minute walking distance on postoperative day 2 and its difference from baseline were comparable between the groups. Similar findings were observed in the age ≥ 50 subgroup.

All dimensions of quality of life assessed by EuroQol Group’s EQ-5D-5L questionnaire including mobility, self-care, usual activities, pain/discomfort, and anxiety were not different between the study groups. Additionally, the visual analogue scores on overall health status were comparable, 80.0 (35.0–100.0) in the intervention group and 80.0 (45.0–100.0) in the control group (*p* = 0.23). These findings remained the same in the age ≥ 50 subgroup.

Incidence of postoperative complications, mainly febrile morbidity was significantly higher in the control group. (Table 5) Of the six participants with febrile morbidity, five resulted from atelectasis and one from pelvic infection. For the participants age ≥ 50, 4 (13.3%) in the control group vs. none in the intervention group had postoperative complications (*p* = 0.04). For the 4 patients with complications, 2 patients had febrile morbidity and 2 patients had bowel ileus. Although there was no significant wound complication documented, visible blood stain on the wound dressing resulting from superficial oozing was observe in 3 participants (8.1%) in the intervention group and 5 participants (16.7%) in the control group (*p* = 0.45). 

## 4. Discussion

When the entire study cohort was considered, the proposed benefits of postoperative abdominal binder use on pain reduction, mobility enhancement, and quality of life improvement could not be clearly demonstrated. The change in pain scores and six-minute walking distance from baseline was comparable between the intervention and the control groups. We observed significantly lower postoperative febrile morbidity in the intervention group. It is important to note, however, that the baseline postoperative pain score in the control group was significantly higher (3.8 vs. 2.4). Although not statistically significant, the larger amount of intraoperative blood loss (400.0 vs. 300.0 mL) and longer operative duration (191.0 vs. 178.5) suggested that participants in the control group might have had more difficult and traumatic surgeries. This difference in the baseline status between the study groups most likely occurred by chance. However, it substantially complicated interpretation of our results.

Of note, when only the participants age ≥ 50 were considered, the baseline surgical characteristics of the two study groups appeared more comparable prior to assigning intervention. These characteristics included incision length, operative blood loss, operative time, and importantly baseline postoperative pain score. Interestingly, we found the association between the binder use and decreased pain scores on postoperative day 1–3 and reduced amount of postoperative consumption of parecoxib and acetaminophen. Furthermore, the longer six-minute walking distance on postoperative day 3 in those who used binder could represent faster physical function recovery. However, one should be cautious when interpreting findings from post-hoc subgroup analysis with rather small sample size.

Although evidence supports early mobilization as an effective measure to prevent complications and encourage recovery following major abdominal surgery, most patients are reluctant to comply due to pain and the concern of breaking the incision [5,7,19]. Traditionally, patients are instructed to use a temporary technique of using their hands or a pillow to splint the incision site when mobilized. Over the past decade, abdominal binder has been commonly offered to women following gynecologic surgery to provide mechanical support for surgical incision. The expected benefit of binder use largely includes pain relief during movement leading to mobility improvement. This combined effect could potentially lead to decreased incidence of postoperative restrictive pulmonary complications (such as atelectasis leading to pneumonia), which accounts for approximately one-fourth of postoperative mortality occurring within one week following major abdominal surgery, as well as bowel ileus and venous thromboembolism [5,20]. In addition, the occurrence of other wound-related complications such as hematoma or seroma could be reduced. Along with these benefits, patients’ sense of welling may improve. Previously, the proposed advantages of post laparotomy abdominal binder use have been evaluated in various surgical settings with conflicting results. (Table S1) There had been no study that specifically addressed the effect of binder use in the unique group of patients with gynecologic cancers.

Postoperative incisional pain has been the main contributing factor for limited mobilization after major open abdominal surgery as the incision moving along with the body by the pull of abdominal wall muscles greatly aggravates pain. This process leads not only to psychological distress but also stasis-related complications including pulmonary atelectasis from shallow breathing, bowel ileus, venous thromboembolism, and febrile morbidity. In principle, abdominal binder could stabilize the abdominal wall and minimize incision movement while the patients are ambulating. Procedure/incision characteristics, anesthesia, postoperative analgesia, co-existing medical conditions, and patients’ perception all play a part in postoperative pain severity [5,7,8,13]. This could explain the inconsistency in the effect of the binder on pain among previous studies. (Table S1) For younger and healthier women who had less complex procedure such as cesarean section, the benefit of the binder use on pain was less noticeable and conflicting [9,10,11,12]. However, with more extensive and complicated procedures performed for gastrointestinal malignancies, the more consistent pain-relieving effect of the binder was observed [5,7]. From the present study addressing women who underwent standard open major abdominal gynecologic oncology procedures mainly through a vertical incision, the clinically significant benefit of abdominal binder use in pain relief over the first few days after surgery was evident but only in those age ≥ 50.

Postoperative mobilization is directly influenced by the extent of physical trauma from surgery and the recovery from it. For an uneventful surgery, the effectiveness of pain control generally determines the ability to mobilize. In the literature, the beneficial effect of binder use on postoperative mobilization was apparent only in the studies that involved extensive procedures with concurrent positive effect on pain relief [5,7]. In Szender et al. [13], where one-third of participants had gynecologic cancer surgery, the only demonstrable benefit of the binder use in term of increased amount of ambulation in the first 24 h was found only in those aged > 50, with vertical incision and complex procedures. However, other benefits of the binder use especially on pain control were not found in that comparably small study with a mixed population. (Table S1) In the present study, the longer six-minute walking distance on postoperative day 3 in the intervention group was evident either when all participants or only those age ≥ 50 were considered. However, for the entire cohort analysis, the higher baseline postoperative pain score in the control group could in fact be the explanatory factor rather than the binder use. Therefore, the potential benefit of the binder on improving postoperative mobility could be supported only for the gynecologic cancer patients age ≥ 50. Of note, the changes in the six-minute walking distance from baseline were not different between the study groups. This could be explained by the inadequate sample size for this outcome. 

Theoretically, improvement in pain control and mobilization would lead to decreased risk of complications. However, any significant effect of abdominal binder use on the incidence of postoperative complications has not been shown in the previous studies. Postoperative pulmonary function, in particular, was examined in many studies without demonstrable positive effect associated with the binder use. In this study, although we found significantly lower incidence of febrile morbidity in the intervention group, it is important to differentiate whether this positive outcome in the intervention group actually was the result of binder use or merely the consequence of the more complicated surgery experienced by the control group especially when all participants was considered. Again, our data only supported the possible benefit of the binder use in this regard only in the age ≥ 50 subgroup. 

The strength of this study was the prospective randomized controlled design with allocation concealment and good participant compliance. However, as noted above, the baseline postoperative pain scores were different between the groups when the entire study cohort was considered. In addition, some patients refused to continue participation after randomization and requested that they exit the project. The main reason for refusal to carry on with the project was personal and mostly related to decreased performance and unwillingness to cooperate with the protocol. Also, because of the nature of the investigation assessed, the participants were not blinded. With subjective outcomes like pain, this could lead to reporting bias. Furthermore, the sample size may not be adequate for detecting small but clinically significant difference in outcomes such as the change in the walking distance from the baseline and some less common postoperative complications. It should also be noted that the results of this study could not be directly generalized to those with ostomy.

## 5. Conclusions

The potential benefits of abdominal binder use in reducing postoperative pain, improving functional recovery, and decreasing postoperative complications after major open abdominal gynecologic cancer surgery could be demonstrated only in the age ≥ 50 subgroup.

## Figures and Tables

**Table 1:** Baseline clinical and surgical characteristics.

Characteristics	Abdominal Binder (*N* = 56)	No Binder (*N* = 53)	*p*-Value
Age, years	55.5 (27.0–80.0)	54.0 (28.0–77.0)	0.87
Body mass index	23.8 (17.8–38.3)	25.3 (13.3–36.0)	0.40
Education Graduate degree Bachelor’s degree Vocational degree Secondary school degree Primary school degree Not attending formal school	5 (8.9) 17 (30.4) 3 (5.4) 6 (10.7) 21 (37.5) 4 (7.1)	5 (9.4) 9 (17.0) 3 (5.7) 6 (11.3) 22 (41.5) 8 (15.1)	0.59
Marital status Single Married Divorced/Widow	12 (21.4) 40 (71.4) 4 (7.1)	4 (7.5) 44 (83.0) 5 (9.4)	0.12
Coexisting medical conditions	29 (51.8)	22 (41.5)	0.28
Incisional type Vertical midline Pfannenstiel Maylard	51 (91.1) 4 (7.1) 1 (1.8)	44 (83.0) 8 (15.1) 1 (1.9)	0.41
Incision length, cms	15.0 (9.0–25.0)	15.0 (10.0–19.0)	0.61
Primary site of cancer Cervix Corpus Ovary	16 (28.6) 21 (37.5) 19 (33.9)	18 (34.0) 19 (35.8) 16 (30.2)	0.73
Stage Early Advanced	40 (71.4) 16 (28.6)	39 (73.6) 14 (26.4)	0.57
Type of anesthesia General General with epidural block	42 (75.0) 14 (25.0)	42 (79.2) 11 (20.8)	0.60
Postoperative intravenous patient-controlled analgesia	8 (14.3)	8 (15.1)	0.91
Postoperative repeated doses of epidural morphine	4 (7.1)	7 (13.2)	0.29
Type of hysterectomy Not done Class I Class II Class III	4 (7.1) 35 (62.5) 2 (3.6) 15 (26.8)	1 (1.9) 33 (62.3) 1 (1.9)18 (34.0)	0.50
Pelvic lymphadenectomy	41 (73.2)	41 (77.4)	0.62
Paraaortic lymphadenectomy	12 (21.4)	12 (22.6)	0.88
Oophorectomy	51 (91.1)	52 (98.1)	0.21
Salpingectomy	53 (94.6)	52 (98.1)	0.62
Omentectomy	32 (57.1)	31 (58.5)	0.89
Appendectomy	11 (19.6)	8 (15.1)	0.53
Intraoperative complication	0 (0.0)	1 (1.9)	0.49
Intraoperative blood loss, mL	300.0 (50.0–1500.0)	400.0 (50.0–2000.0)	0.09
Duration of operation, minutes	178.5 (95.0–291.0)	191.0 (89.0–287.0)	0.30

Data expressed as median (range) or numbers (percentage).

**Table 2:** Postoperative pain scores.

Assessment Time	Abdominal Binder	No Binder	*p*-Value
**Entire study**	**(*N* = 56)**	**(*N* = 53)**	
Baseline	2.4 (0.0–6.5)	3.8 (0.0–7.5)	0.02 *
Day 1	3.4 (0.1–7.3)	4.6 (0.1–8.8)	0.01 *
Difference between Day 1 and baseline	0.7 (−5.6 to 7.3)	0.7 (−2.8 to 7.8)	0.98
Day 2	1.8 (0.0–7.7)	2.9 (0.0–8.5)	0.03 *
Difference between day 2 and baseline	−0.2 (−5.4 to 7.7)	−0.7 (−4.8 to 6.0)	0.55
Day 3	1.1 (0.0–5.3)	2.2 (0.0–6.9)	0.13
Difference between day 3 and baseline	−0.8 (−5.1 to 4.3)	−1.0 (−6.0 to 5.5)	0.80
**Age ≥ 50 subgroup**	**(*N* = 37)**	**(*N* = 30)**	
Baseline	2.6 (1.9)	3.2 (2.4)	0.21
Day 1	3.4 (2.1)	4.8 (2.1)	0.01 *
Difference between Day 1 and baseline	0.9 (2.0)	1.6 (2.6)	0.19
Day 2	2.2 (1.6)	3.8 (2.0)	<0.01 *
Difference between day 2 and baseline	−0.4 (2.0)	0.6 (2.6)	0.08
	**(*N* = 19)**	**(*N* = 21)**	
Day 3	1.4 (1.3)	3.1 (2.3)	0.01 *
Difference between day 3 and baseline	−1.2 (1.8)	0.4 (3.0)	0.05 *

Data expressed as median (range) or mean (SD), * Statistically significant.

**Table 3:** Postoperative pain medication consumption.

Post-op Consumptions	Allocations		*p*-Value
	Abdominal Binder	No Binder	
**Entire study**			
Morphine Total, mg	6 (3–6)	5 (3–6)	0.79
Parecoxib Total, mg	40 (40–80)	80 (40–80)	0.03 *
Acetaminophen Total, mg	1000 (500–1000)	1500 (1000–2000)	0.01 *
Tramadol Total, mg	50 (50–50)	50 (50–50)	0.41
Diclofenac Total, mg	1 (1–1)	50 (25–75)	0.22
**Age ≥ 50 subgroup**			
Morphine Total, mg	3 (3–6)	3 (3–6)	0.91
Parecoxib Total, mg	40 (40–40)	80 (40–80)	0.01 *
Acetaminophen Total, mg	1000 (500–1000)	1500 (1000–2000)	<0.01 *
Tramadol Total, mg	50 (50–50)	50 (50–50)	-
Diclofenac Total, mg	-	25 (25–25)	0.32

Data expressed as median (range), * Statistically significant.

**Table 4:** Six-minute walk test outcomes.

Assessment	Abdominal Binder	No Binder	*p*-Value
**Entire study**	**(*N* = 56)**	**(*N* = 53)**	
Baseline six-minute walking distance, meters	313.0 (150–484)	309.0 (172–660)	0.94
Day 2 six-minute walking distance, meters	161.5 (58–380)	180.0 (28–360)	0.41
Day 3 six-minute walking distance, meters	232.5 (92–420)	197.0 (73–340)	0.04 *
Distance difference (between day 2 and baseline), meters	−124.5 (−343 to 59)	−137.0 (−472 to −15)	0.19
Distance difference (between day 3 and baseline), meters	−71.0 (−214 to 270)	−115.0 (−540 to 49)	0.07
**Age ≥ 50 subgroup**	**(*N* = 37)**	**(*N* = 30)**	
Baseline six-minute walking distance, meters	311.0 (150–484)	300.0 (172–660)	0.54
Day 2 six-minute walking distance, meters	150.0 (58–380)	154.5 (60–360)	0.72
Day 3 six-minute walking distance, meters	211.0 (92–420)	182.0 (80–340)	0.04 *
Distance difference (between day 2 and baseline), meters	−129.0 (−320 to 59)	−141.5 (−465 to −20)	0.50
Distance difference (between day 3 and baseline), meters	−76.0 (−214 to 270)	−115.0 (−540 to 2)	0.11

Data expressed as median (range), * Statistically significant.

**Table 5:** Postoperative complications.

Complications	Abdominal Binder (*N* = 56)	No Binder (*N* = 53)	*p*-Value
All complications	0 (0.0)	8 (15.1)	<0.01*
Febrile morbidity	0 (0.0)	6 (11.3)	0.01 *
Clinically significant ileus	0 (0.0)	2 (3.8)	0.23
Wound complication	0 (0.0)	0 (0.0)	-

Data expressed as numbers (percentage), * Statistically significant.

## Data Availability

The data presented in this study are available on request from the corresponding author.

## Supplementary Materials

The following are available online at https://www.mdpi.com/article/10.3390/medicina57050481/s1. Table S1 Randomized controlled trials that examined the effectiveness of abdominal binder following major open abdominal surgery.

## References

[1] Scott R., Hawarden A., Russell B., Edmondson R.J. (2020). Decision-Making in Gynaecological Oncology Multidisciplinary Team Meetings: A Cross-Sectional, Observational Study of Ovarian Cancer Cases. Oncol. Res. Treat..

[2] Garg P.K., Kumar R., Choudhary D. (2021). Cytoreductive or debulking surgery in ovarian cancer: The name does matter!. J. Surg. Oncol..

[3] Tozzi R., Ferrari F., Nieuwstad J., Campanile R.G., Soleymani Majd H. (2020). Tozzi classification of diaphragmatic surgery in patients with stage IIIC-IV ovarian cancer based on surgical findings and complexity. J. Gynecol. Oncol..

[4] Rothman J.P., Gunnarsson U., Bisgaard T. (2014). Abdominal binders may reduce pain and improve physical function after major abdominal surgery—A systematic review. Dan. Med. J..

[5] Cheifetz O., Lucy S.D., Overend T.J., Crowe J. (2010). The effect of abdominal support on functional outcomes in patients following major abdominal surgery: A randomized controlled trial. Physiother. Can..

[6] Larson C.M., Ratzer E.R., Davis-Merritt D., Clark J.R. (2009). The effect of abdominal binders on postoperative pulmonary function. Am. Surg..

[7] Arici E., Tastan S., Can M.F. (2016). The effect of using an abdominal binder on postoperative gastrointestinal function, mobilization, pulmonary function, and pain in patients undergoing major abdominal surgery: A randomized controlled trial. Int. J. Nurs. Stud..

[8] Olsen M.F., Josefson K., Wiklund M. (2009). Evaluation of abdominal binder after major upper gastrointestinal surgery. Adv. Physiother..

[9] Chankhunaphas W., Charoenkwan K. (2020). Effect of elastic abdominal binder on pain and functional recovery after caesarean delivery: A randomised controlled trial. J. Obstet. Gynaecol..

[10] Ghana S., Hakimi S., Mirghafourvand M., Abbasalizadeh F., Behnampour N. (2017). Randomized controlled trial of abdominal binders for postoperative pain, distress, and blood loss after cesarean delivery. Int. J. Gynaecol. Obstet..

[11] Gillier C.M., Sparks J.R., Kriner R., Anasti J.N. (2016). A randomized controlled trial of abdominal binders for the management of postoperative pain and distress after cesarean delivery. Int. J. Gynaecol. Obstet..

[12] Gustafson J.L., Dong F., Duong J., Kuhlmann Z.C. (2018). Elastic abdominal binders reduce cesarean pain postoperatively: A randomized controlled pilot trial. Kans. J. Med..

[13] Szender J.B., Hall K.L., Kost E.R. (2014). A randomized-clinical trial examining a neoprene abdominal binder in gynecologic surgery patients. Clin. Exp. Obstet. Gynecol..

[14] Nelson G., Bakkum-Gamez J., Kalogera E., Glaser G., Altman A., A Meyer L., Taylor J.S., Iniesta M., LaSala J., Mena G. (2019). Guidelines for perioperative care in gynecologic/oncology: Enhanced Recovery After Surgery (ERAS) Society recommendations-2019 update. Int. J. Gynecol. Cancer.

[15] Solway S., Brooks D., Lacasse Y., Thomas S. (2001). A qualitative systematic overview of the measurement properties of functional walk tests used in the cardiorespiratory domain. Chest.

[16] American Thoracic Society (2002). ATS statement: Guidelines for the six-minute walk test. Am. J. Respir. Crit. Care Med..

[17] Herdman M., Gudex C., Lloyd A., Janssen M., Kind P., Parkin D., Bonsel G., Badia X. (2011). Development and preliminary testing of the new five-level version of EQ-5D (EQ-5D-5L). Qual. Life Res..

[18] Jensen M. (2003). Interpretation of visual analog scale ratings and change scores: A reanalysis of two clinical trials of postoperative pain. J. Pain.

[19] Havey R., Herriman E., O’Brien D. (2013). Guarding the gut: Early mobility after abdominal surgery. Crit. Care Nurs. Q..

[20] Arozullah A.M., Conde M.V., Lawrence V.A. (2003). Preoperative evaluation for postoperative pulmonary complications. Med. Clin. N. Am..

