# Supplementary material for: Effect of Elastic Abdominal Binder on Pain and Functional Recovery Following Gynecologic Cancer Surgery: A Randomized Controlled Trial
Source: Medicina (Kaunas). 2021 May 12;57(5):481. doi: 10.3390/medicina57050481 (PMC8151580; doi:10.3390/medicina57050481)
Supplement: Supplementary file 1 [file medicina-57-00481-s001.zip › medicina-1192674-supplementary.pdf]

Table S1 Randomized controlled trials that examined the effectiveness of abdominal binder following major open abdominal surgery

Studies	Patients' condition/ Surgical procedures	N (Analyzed)	Binder use protocol	Pain	Physical function	Psychological distress/ Quality of life	Pulmonary function	Other postoperative complications
**Gastrointestinal surgery**								
Olsen *et al*, 2009 [8]	Upper gastrointestinal conditions/ Pancreatic surgery, gastrectomy, miscellaneous upper gastrointestinal surgery	37<br>Binder 18<br>No binder 19	Wore as much as possible during the first 5 days	-	-	NA	-	NA
Cheifetz *et al*, 2010 [5]	Gastrointestinal malignancies, inflammatory bowel disease/<br>Whipple's procedure, colectomy, abdominoperineal resection, splenectomy, pancreatectomy	60<br>Binder 30<br>No binder 30	Starting POD 1, wore at all times when out of bed for 5 days	+<br>Unchanged after surgery in binder group, increased significantly in no binder group	+<br>Similar 6MWT distances for both groups on POD 3 and 5, but greater improvement in 6MWT distance on POD 5 in binder group	+<br>Unchanged after surgery in binder group, increased significantly in no binder group	-	NA
Arici *et al*, 2016 [7]	Gastrointestinal conditions/ Colectomy, gastrectomy, Whipple's procedure, low anterior resection, abdominoperineal resection, hepatectomy	84<br>Binder 42<br>No binder 42	Wore at all times when out of bed starting with the first mobilization	+<br>Lower sensory sub-scale, total, and VAS pain scores on POD 1, 4, and 7 in binder group	+<br>Longer 6MWT distances on POD 4 and 7 in binder group	NA	-	-<br>Comparable rate of nausea/vomiting and return of bowel function
**Cesarean section**								
Gillier *et al*, 2016 [11]	Pregnancy/Cesarean section through transverse skin incision with regional anesthesia	155<br>Binder 87<br>No binder 68	Started before leaving operating room.<br>Encouraged to	-	NA	-	NA	NA

			wear at all time, unmeasured breaks allowed					
Ghana *et al*, 2017 [10]	Pregnancy/Cesarean section through transverse Pfannenstiel skin incision with regional anesthesia	178<br>Binder 89<br>No binder 89	Started 2 hours after delivery, used for 2 days. Released from 10 pm to 8 am	+<br>Lower VAS pain scores at all time points assessed at 6 hours interval in binder group	NA	+<br>Lower (better) SDS scores at 24 and 48 hours after surgery in binder group	NA	NA
Gustafson *et al*, 2018 [12]	Pregnancy/Cesarean section with regional anesthesia	56<br>Binder 29<br>No binder 27	Started immediately postoperative, wore for the first 24 hours	+<br>Lower pain scores at 24 hours after surgery in binder group	NA	NA	NA	NA
Chankhunaphas *et al*, 2020 [9]	Pregnancy/Cesarean section	179<br>Binder 89<br>No binder 90	Started before leaving operating room. Encouraged to wear at all time, periods of break allowed at convenience	-	-	-<br>Better score in the self-care dimension of the EQ-5D-5L in no binder group	NA	-
**Gynecologic surgery**								
Szender *et al*, 2014 [13]	Gynecologic conditions<br>Uterine leiomyoma 40%<br>Gynecologic cancer 37%<br>Others 24%	75<br>Binder 36<br>No binder 39	Started before leaving operating room, wore for the first 24 hours	-	±<br>Increased number of ambulatory events in the first 24 hours in	NA	NA	NA

					binder only in vertical incision and age ≥ 50 subgroups			

Abbreviation POD: postoperative day, 6MWT: six-minute walk test, VAS: visual analog scale, SDS: symptom distress scale
Symbol + : improved with binder use - : not improved with binder use NA: not assessed
